# Life-threatening bleeding in a patient with pemphigoid-induced acquired hemophilia A and successfully treated with rituximab and rFVIIa: A case report

**DOI:** 10.1097/MD.0000000000024025

**Published:** 2021-01-22

**Authors:** Hongbing Ma, Hong Chang

**Affiliations:** Department of Hematology, West China Hospital, Sichuan University, Chengdu 610041, China.

**Keywords:** acquired hemophilia A, pemphigus, rFVIIa, rituximab

## Abstract

**Rationale:**

Acquired hemophilia A (AHA) is a rare bleeding disorder with prolonged activated partial thromboplastin time (aPTT). Severe hemorrhage may occur, especially in refractory AHA.

**Patient concerns:**

We reported a 63-year-old man who suffered from life-threatening bleeding after the onset in lower limbs.

**Diagnoses:**

The patient was diagnosed as AHA which was related to pemphigoid.

**Interventions:**

The patient had no response to the first-line treatment with corticosteroid and cyclophosphamide. Meanwhile, fatal hemorrhage occurred successively in thoracic cavity and right frontal lobe. rFVIIa and rituximab were administered.

**Outcomes:**

The patient survived from the life-threatening hemorrhage with a normal aPTT. His aPTT and FVIII:C level was normal during the follow-up of 6 months.

**Lessons:**

Rituximab and rFVIIa can play a critical role in rescuing AHA that is refractory to the first-line treatment.

## 1 Introduction

Acquired hemophilia A (AHA) is a rare bleeding disorder with an incidence of approximately 1.5 cases/million/year,^[1]^ which is characterized by autoantibodies directed against circulating coagulation factor (F) VIII. Typically, patients have no prior history of a bleeding disorder and present with spontaneous bleeding and an isolated prolonged activated partial thromboplastin time (aPTT). The incidence of fatal bleeding in acquired hemophilia patients is high, ranging between 22%^[1]^ and 31%^[2]^ in older reports when therapeutic options for acute bleeding were limited, and 9% in a more recent study.^[3]^ Management of this clinical entity is still challenging. We reported a patient with AHA secondary to pemphigus who responded poorly to the corticosteroids in combination with cyclophosphamide treatment but responded well to the treatment with rituximab in conjunction with low-dose corticosteroids.

## 2 Case report

**Figure 1:**
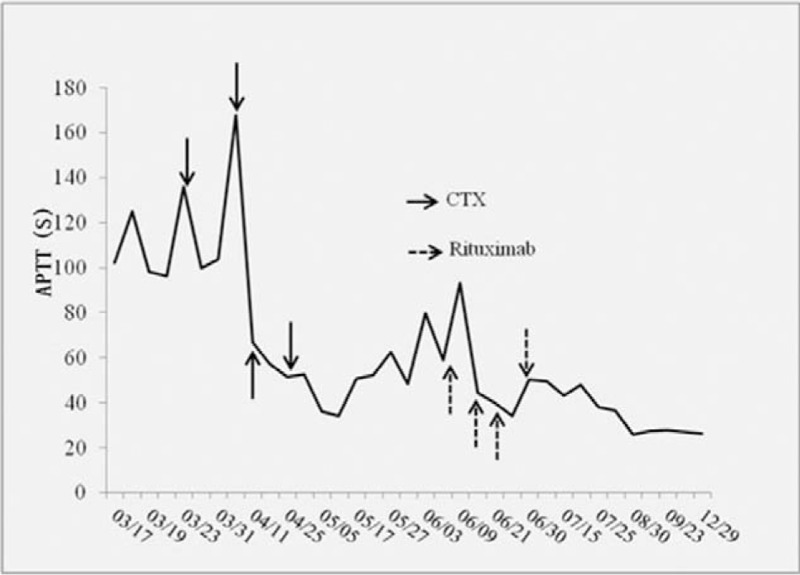
Trend of changes in activated partial thromboplastin time (aPTT). The arrow with full line indicated the use of cyclophosphamide (CTX), while the arrow with dotted line indicated the use of rituximab. The aPTT returned to normal range eventually.

**Figure 2:**
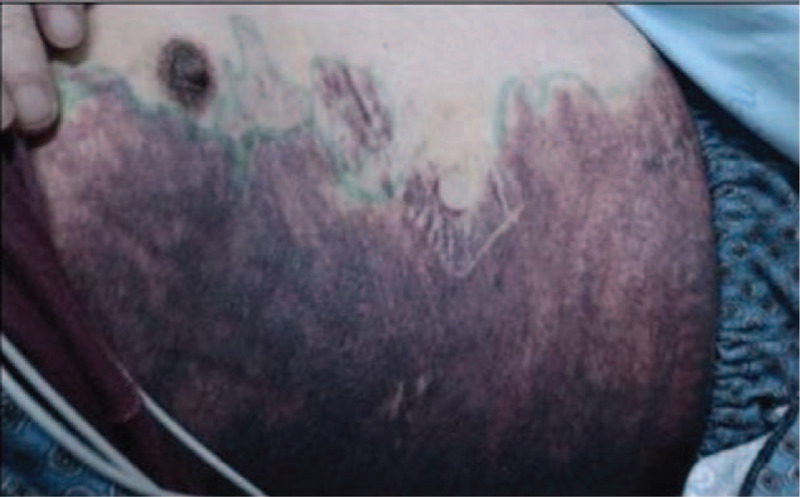
Large area of ecchymosis was noted on the right flank.

**Figure 3:**
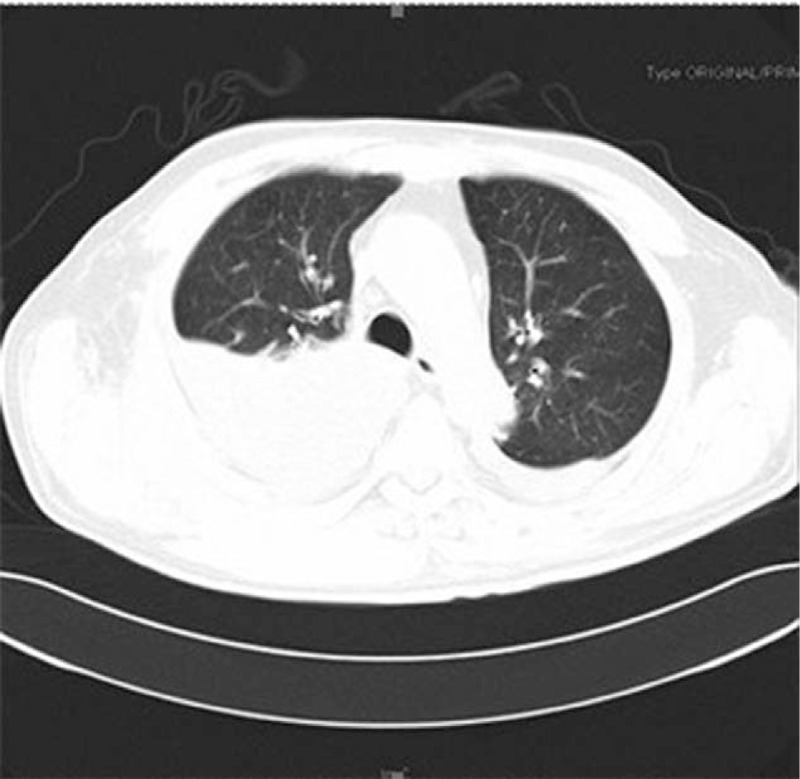
Computed tomography (CT) scan of chest revealed right-sided pleural effusion.

**Figure 4:**
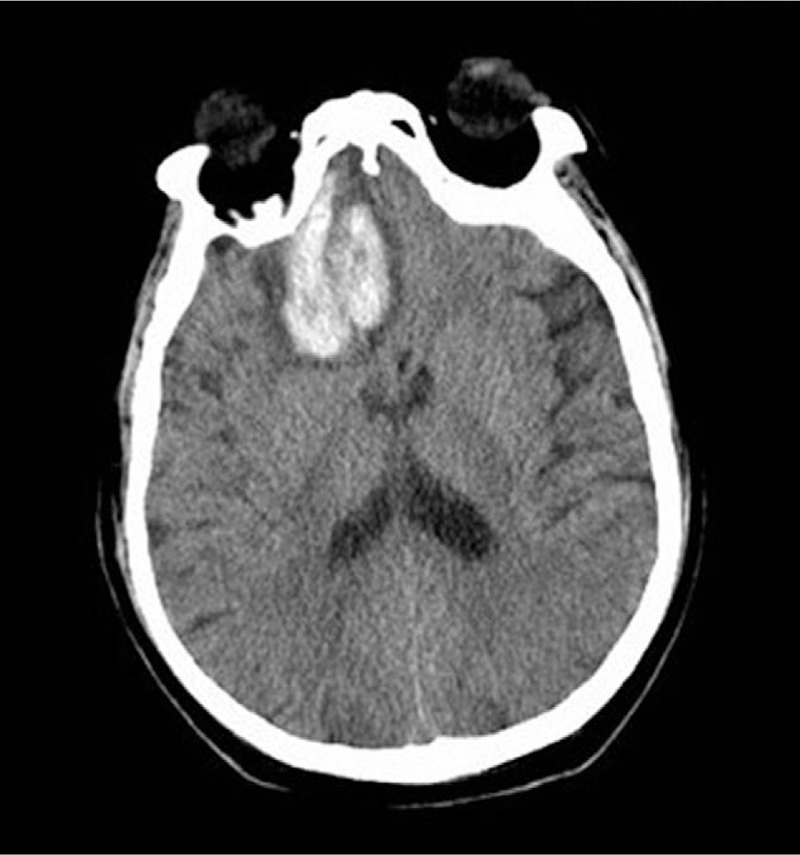
Cerebral CT revealed hematoma in the area of right frontal lobe.

A 63-year-old man was referred to our hospital because of ecchymosis, pain, and swelling of the lower limbs. The patient was diagnosed with pemphigoid 7 months before admission when he complained of erythema, water blister, and pruritus. He initially received methylprednisolone and was then switched to prednisone and tapered with clinical benefit. The medicine was stopped with remission of the symptoms shy of hemorrhage. There was no history of previous bleeding episodes. His family history was unremarkable. Physical examination revealed anemic signs, sporadic pigmentation in upper and lower limbs, and large ecchymosis at the area of right lower quadrant of abdomen, right buttock, and the swollen lower limbs. Neither hepatomegaly nor splenomegaly nor water blister was detected. Complete blood count revealed mild thrombocytopenia (platelet 93 × 10^9^/L) and moderate anemia (hemoglobin, Hb, 65 g/L). His blood chemistry revealed elevated liver function parameters (ALT 66 IU/L, reference <38 IU/L; AST 118 IU/L, reference <37 IU/L; LDH 486 IU/L, reference 110–220 IU/L). Anti-dsDNA, anti-neutrophil cytoplasmic antibody (ANCA), anti-cardiolipin antibody (ACA) were all unremarkable, while C3 and C4 were decreased (C3 0.33 g/L, reference 0.785–1.52 g/L; C4 0.059 g/L, reference 0.145–0.36 g/L). Serum levels of tumor markers (AFP, CEA, CA19-9, and CA125) were within normal limit and PET-CT did not reveal any remarkable findings. The aPTT was prolonged (102.1 s, reference 20–40 s), when mixing with normal plasma at a ratio of 1:1, the aPTT was 47.4 s and turned to be 78.2 s after 1 to 2 incubation at 37°C, while the prothrombin time (PT) and fibrinogen (FIB) were within normal limit (PT 10.5 s, reference 9.6–12.8 s; FIB 3.55 g/L, reference 2.0–4.0 g/L). The activity of factor VIII decreased remarkably (2.7%, reference 60–150%); the activity of factor IX, XI was within normal limit (F IX: C 68.2%, reference 60–150%; FXI: C 94.1%, reference 60–150%); and von Willebrand factor antigen (vWF:Ag) was 1.864 times of the normal value. Therefore, a diagnosis of acquired hemophilia A was made and the patient then received an intravenous F VIII, followed by 10 mg of dexamethasone daily and 1.0 g of cyclophosphamide once a week and red blood cells transfusion. The aPTT firstly rose to 167.7 s then gradually decreased (Fig. 1). On the 25th day after admission, when initial clinical improvement was observed and Hb was104 g/L, the patient suddenly developed dizziness, dyspnea, hypotension (BP: 75/55 mm Hg), large ecchymosis on the right flank (Fig. 2), and large hematoma in the region of right shoulder. New laboratory tests showed Hb of 59 g/L, and the aPTT had decreased to 66.9 s. The patient then received blood transfusion, factor VIII, prothrombin complex concentrates, anti-fibrinolysis but dyspnea was not relieved. Computed tomography (CT) was then performed and showed right-sided pleural effusion (Fig. 3). Intravenous rFVIIa was immediately given at a dose of 6 mg and a satisfactory control on bleeding was achieved. On the 48th day after admission when the aPTT then was 52.8 s, inhibitor titer was 13 BU, the patient suddenly complained of frontal headache. Cerebral CT revealed hematoma (size: 4.9 cm × 3.1 cm) at the area of right frontal lobe (Fig. 4). The patient was treated with mannitol, furosemide, and intravenous rFVIIa at a dose of 6 mg each time (twice, 12 mg in total). This controlled the hemorrhage and the symptomatic improvement was observed. On the 69th day after admission, the patient complained of fever and productive cough. A diagnosis of agranulemia combined with infection was established based on the laboratory and microbiologic findings. Therefore, the patient was given rituximab (375 mg/m^2^) once a week (4 times in total), accompanied with anti-infection therapy and the dose of corticosteroid was rapidly tapered off. The aPTT gradually decreased with improvement of signs and symptoms and the patient was discharged from our hospital with 15 mg of prednisone as well as with a treatment plan of a slow tapering of steroid. His aPTT and FVIII:C levels were normal during the follow-up of 6 months.

## 3 Discussion

AHA patients may present initially to physicians from specialties, who may not have experience with this rare disorder, leading to a delayed or missed diagnosis. Therefore, any acute or recent onset of bleeding symptoms in a patient, especially a senile or post-partum patient, with no previous history of bleeding, and an unexplained isolated prolonged aPTT may be investigated for AHA. Mixing tests are customarily performed to distinguish between factor deficiency and the presence of an inhibitory substance. Since FVIII inhibitors are time and temperature-dependent, mixing studies performed immediately and after 2 h of incubation should be compared. Compared to an immediate mixture, prolongation of the aPTT in the mixture plasma of patient and healthy donor after a 1 to 2 h incubation is typical for FVIII autoantibodies. Immediate correction of the aPTT with normal plasma does not exclude AHA, prolongation may occur after incubation (1–2 h) at 37°C. The aPTT of our patient, for example, was 102.1 s at admission. When mixing with normal plasma at a ratio of 1:1, the aPTT was 47.4 s and turned to be 78.2 s after 1 to 2 incubation at 37°C. Furthermore, FVIII, IX, XI, and XII levels should be measured in patients with a prolonged aPTT and with clinical feature suggesting AHA. An isolated low FVIII level and uncorrectable aPTT are suggestive of AHA. The Bethesda assay was developed to quantify FVIII autoantibodies. For laboratory reasons, the patient did not undergo the inhibitor titer on admission. However, it was 13 BU after treatment. Control of acute bleeding in AHA is generally the immediate priority. The incidence of fatal bleeding in acquired hemophilia patients is high. Death within the first week is caused by gastrointestinal and lung bleeding, and later deaths are predominantly due to intracranial and retroperitoneal hemorrhages.^[1,2]^ The correlation between laboratory assessments and bleeding phenotype in AHA has recently been recognized, but remains poorly understood .^[1,4]^ When the fatal bleeding occurred in our patient, the aPTT had decreased from 167.7 s to 52.8 to 66.9 s.Therefore, we highly recommend that frequent clinical assessment, monitoring of the Hb, and radiological imaging are often the more reliable indicators of significant bleeding.

The international recommendations recommend the use of rFVIIa or aPCCs for the treatment of severe bleeding in patients with AHA and suggest bolus injection of rFVIIa at 90 mcg/kg every 2 to 3 h until hemostasis is achieved.^[5]^ Patients diagnosed with AHA should undergo immediate inhibitor eradication to restore normal hemostasis. Corticosteroids, either alone or in combination with cyclophosphamide are suggested especially if the patient has already been treated with corticosteroids for other medical conditions. If no response is observed after 4 to 6 weeks, treatment with rituximab alone or in combination with corticosteroids is an alternative. Rituximab showed efficacy both as first-line therapy in patients with a recent diagnosis, and as rescue therapy in patients unresponsive to other immunosuppressive treatments.^[6]^ Most immunosuppressive drugs are associated with side effects, including neutropenia-related infections and sepsis. Because the majority of AHA patients are elderly and likely to have concomitant medical conditions, treatment regimens should aim to balance the need to eradicate the inhibitor quickly, thereby reducing the risk of severe bleeding episodes, and the time and exposure to the side effects of immunosuppressive therapy. The dose of corticosteroid therapy should be rapidly tapered off after successful therapy or after a switch to second-line treatment.^[7]^

The etiology of acquired hemophilia A remains unclear. In approximate half of cases, factor VIII autoantibodies occur in patients without any identifiable cause, while the remaining cases may be associated with autoimmune diseases, infections, use of medications in the post-partum period, or underlying hematological or solid tumors.^[8]^ The most common associated illnesses are autoimmune diseases, such as pemphigoid in our patient. Pemphigoid is an autoimmune skin disease with antibodies against the cutaneous basement membrane. The disordered immunity may also generate antibody against factor VIII as it is reported in systemic lupus erythematosus.^[9,10]^

In summary, we describe a patient with pemphigoid who subsequently developed AHA with life-threatening bleeding and extensive side effects of immunosuppressive therapy. Rituximab eradicated the inhibitory autoantibody and combination with rFVIIa successfully rescued the life-threatening bleeding.

## Author contributions

**Formal analysis:** Hongbing Ma.

**Investigation:** Hong Chang.

**Writing – original draft:** Hongbing Ma.

**Writing – review & editing:** Hong Chang.

**Formal analysis:** Hongbing Ma.

**Project administration:** Hong Chang.

**Writing – original draft:** Hongbing Ma.

**Writing – review & editing:** Hong Chang.
